# Supplementary material for: Grains, trade and war in the multimodal transmission of Rice yellow mottle virus: An historical and phylogeographical retrospective
Source: PLoS Pathog. 2025 Jun 17;21(6):e1013168. doi: 10.1371/journal.ppat.1013168 (PMC12173394; doi:10.1371/journal.ppat.1013168)
Supplement: Attachment — Submitted filename: Rebuttal letter 250219.docx [file ppat.1013168.s007.docx]

PPATHOGENS-D-24-01977

Grains, trade and war in the multimodal transmission of Rice yellow mottle virus: an historical and phylogeographical retrospective

PLOS Pathogens

Dear Editors and Reviewers,

Many thanks for your valuable advice and the opportunity to revise the paper for resubmission. In the enclosed version, we have responded to all the comments as requested.

**Editor Comments :**

*Thank you for the submission of this interesting manuscript. I am returning it with three reviews. The three reviewers all find the study to be of notable interest to a general audience and describe it as "compelling" and "a major advance". I concur with these assessments. There are no major concerns with the analytical methods that would require additional work but a few points to discuss further. Importantly, all three reviewers indicate that the manuscript would be significantly stronger with improvements to the text and figures.*

*Please be attentive to all of the comments from the reviewers. It will be especially important to address the following points about figures from reviewer 1 and myself and about the text as summarized from reviewers 1-3. These are improvements that will strengthen your paper and clarify important points for the readers.*

**Figure points:**

*1. Can figures 1 & 2 be annotated and/or combined in some way, as reviewer 1 suggests, to increase their value to the reader?*

Figure 2 was removed as a phylogeny reconstructed from full-length genomic sequences of isolates representative of the genetic and geographic diversity is also provided in Figure 8b (now Figure 5). The insertions and deletions are now indicated in Figure 1. The amino-acids involved (and not + or -), their ORF and their position are indicated.

*2. Are all of figures 3-6 essential? Can they be annotated/edited to support better comprehension? Is it possible to specify directionality to the lines as arrows? Please test the revised figures with a general scientific audience before re-submission.*

One of the co-author of the article, François Chevenet, implemented new functionalities in the software programme EvoLaps (including the directionality specified by arrows). This explains the delay in the submission of the revised version. This new version of EvoLaps (https://[www.evolaps.org/evolaps3.14/](http://www.evolaps.org/evolaps3.14/)) was used to generate improved figures that depict the dynamics of dispersal of the RYMV. This explains the delay in the submission of the revised version.

We propose to keep figure 6 (renamed figure 3). As far as we know, this is a rare instance (possibly unique in plant virology) where the same phylogeographic scenario is obtained when reconstructed from different portions of the genomes. This strengthens the interpretation of the phylogeography of RYMV in East Africa. With other plant viruses, partially contradictory phylogeographic scenarios are obtained from different portions of the genomes. See for instance the following references:

>Kawakubo S, Gao F, Li S, Tan Z, Huang YK, Adkar-Purushothama CR, Gurikar C, Maneechoat P, Chiemsombat P, Aye SS, Furuya N, Shevchenko O, Špak J, Škoríc D, Ho SYW, Ohshima K (2021). Genomic analysis of the brassica pathogen turnip mosaic potyvirus reveals its spread along the former trade routes of the Silk Road. Proceedings of the National Academy of Sciences the United States of America, 118(12), e2021221118.

>S Wei, G Chen, H Yang, L Huang, G Gong, PG Luo, Min Zhang. 2023. Global molecular evolution and phylogeographic analysis of barley yellow dwarf virus based on the cp and mp genes. Virology Journal, 20:130. https://doi.org/10.1186/s12985-023-02084-1

>Zhen He, Wen Chen, Ryosuke Yasaka, Chunfeng Chen, Xijun Chen. 2019. Temporal analysis and adaptive evolution of the global population of potato virus M. Infection, Genetics and Evolution 73 (2019) 167–174.

Figures 3-4-5 have been combined into a single composite figure (renamed figure 2) and annotated to support better comprehension. A time-calibrated phylogenic tree has been introduced to better visualize what period of the evolutionary history is mapped and what stage of the lineage diversification is involved. We now also distinguish ongoing movements (lines in black) from completed ones (gradient of colors). Arrows are used to visualize the movements. A more detailed geographical map layer is now used. The revised figures took advantage of recent developments, now publicly available, of the phylogeographic software Evolaps version 3 (<https://www.evolaps.org/evolaps3.14/>).

Figure 9 (renamed figure 6) has been edited with new tools in Evolaps (see above). A time calibrated phylogenic tree has been introduced

*3. Reviewer 1 found figures 7 and 9 to be most helpful, and I appreciated figure 8. Please consider whether these also can be "tuned up" further for maximal comprehension.*

Figure 8 (renamed figure 5) supports the hypothesis that there was a single successful introduction of RYMV from East to West Africa. It is based on the analysis of the full genome, taking or not the putative recombination events into account through network and phylogenetic trees, respectively. It is a necessary prerequisite to the phylogeographic reconstruction of RYMV throughout Africa in Figure 9 (renamed figure 6) based on the coat protein gene that showed the dispersal from East Africa towards West Africa. This is why we propose to keep Figure 8 (renamed figure 5) as a plain figure rather than as a supplementary figure.

Reviewer 1

*Most of the figures are very uninformative in their current form and require much more annotation to convey useful information.*

Figures 3, 4 and 5 have been combined into a single figure (renamed figure 2) to which annotation has been added to convey the necessary information as indicated above.

*Figures 3, 4, 5, 6 and 8 seem like they are attempting to convey the same result but are unclear and uninformative.*

Please see above the answer to the editor for figures 3,4,5 (renamed figure 2) and 6 (renamed figure 3).

Please see above the answer to the editor for figure 8 (renamed figure 5).

*It would be really helpful to illustrate the seven periods that you have identified into a figure of sorts, such as a time line or similar.*

Figures 3-4-5 (renamed figure 2) have been combined into a single and annotated composite figure (see above). Each of the seven periods that were identified and discussed are now distinguished. A time-calibrated phylogenic tree with a time line has been introduced to the figure. A gradient of colors (coordinated between the map and the phylogenetic tree) is now applied to distinguish the different phases of the movement.

Reviewer 3

*The paper largely neglects uncertainty about the origins and movement of RYMV. This is particularly notable for the reconstructed history of events introduced on pages 10 and 11. It would be good to report measures of confidence/uncertainty surrounding the reconstructed ancestral locations and dispersal pathways in both the text and the figures.*

The uncertainty associated with the Bayesian phylogeographic inference is now reported by shaded polygons representing the 80% highest posterior density (HPD) regions for each successive time slice. The HPD polygons at different periods are distinguished by different colors to make them more readable, they correspond to the lineages of the phylogenetic tree and the movement of the phylogeographic reconstructions.

**Text**
Introduction:
*The first paragraph provides essential information for the final conclusions but on first read may not appear fully connected to the rest of the text. Can you please provide a little more context and connection here?*
The following sentences were added at the end of the first paragraph (lines 88-93): “This study aimed to evaluate the relative importance of abiotic and vector-mediated transmission in plant virus species with a dual mode of transmission (i.e. vector and abiotic). We hypothesized that the contribution of abiotic transmission to plant virus spread has been substantially underestimated (Jones 2018; Peters et al, 2024). Rice yellow mottle virus (RYMV), which affects rice crops in Africa, is as an appropriate model to test this hypothesis”.

Discussion:
*A common theme is a need to tighten the Discussion text (reduce repetition) and give it more narrative shape.*

Parts of the paragraphs of the Discussion dealing with methodological issues (sample bias, flexibility of the RRW…) were inserted in the Result (lines 187-191, 235-253, 330-333) or in Materials and Method sections (lines 654-655). A paragraph on the hypothetical role of positive selection in the differential epidemiological success of the strains, a topic that is outside of the scope of this study, has been omitted in order not to dilute the message (lines 474-485). A few paragraphs were shortened.

*In doing so, please address point 6 from reviewer 2 about the historical information, and points 1 and 2 that reviewer 3 raised under major issues (uncertainty about origins/movement, lack of temporal signal).*

The following sentence, directly inspired by the comment, has been added at the beginning of the Discussion (lines 395-396) « The historical data provided circumstantial evidence that support, explain and complement the interpretation of the phylogeographic reconstructions ».

*Reviewer 3's points might be alternatively addressed in methods. Some of the discussion requested is present in the text but may need further highlighting so that readers more readily find it.
Please further consider reviewer 1's suggestion of a timeline figure to illustrate the seven periods you have identified.*
This point has been addressed above in the « Figure points » section, and the figure has been modified accordingly.

*Please further consider reviewer 1's suggestion of a timeline figure to illustrate the seven periods you have identified.*

A dated phylogenetic tree has been added as an inset to the figures indicating the time span for the different periods.

*Finally, please consider the minor points presented by all three reviewers and respond as you see fit to best polish the manuscript quality.*

 See below

**Part II – Major Issues: Key Experiments Required for Acceptance**
Please use this section to detail the key new experiments or modifications of existing experiments that should be absolutely required to validate study conclusions.

*Reviewer #1: While the analysis is robust and I trust the methodology used to obtain these results, the way it is presented is the weakest part of this manuscript and doesn't do the paper justice. Most of the figures are very uninformative in their current form and require much more annotation to convey useful information. For example, Figures 1 and 2 could be combined into a single figure, and it would be useful to illustrate the congruence between them (i.e. using a tanglegram or similar). At the very least, some highlighting of clades and their position on the two trees would be helpful. As well, the + and - symbols could easily be replaced by showing the actual insertions and deletions for context. Figures 3, 4, 5, 6 and 8 seem like they are attempting to convey the same result but are unclear and uninformative. Figures 7 and 9 are the only ones that I feel provide enough useful information and are of adequate quality for a publication. The authors need to revise their presentation and have more consistency throughout to convey the story.*

All the queries regarding the figures have been addressed above, in the section « Figure points ».

*Reviewer #2: No major issue.*

*Reviewer #3: 1) The paper largely neglects uncertainty about the origins and movement of RYMV. This is particularly notable for the reconstructed history of events introduced on pages 10 and 11. It would be good to report measures of confidence/uncertainty surrounding the reconstructed ancestral locations and dispersal pathways in both the text and the figures. Similarly, phylogeographic reconstructions are well known to be highly susceptible to sampling biases. This raises the question of whether the history reconstructed here simply mirrors the geographic distribution of samples. Providing at least some discussion of these issues is important, especially for readers who may be less familiar with the limitations of these phylogeographic methods.*

A single synthetic figure that superimposes the different periods of spread (ex figure 3) was not sufficiently clear to fully distinguish the different periods (despite gradients of colors, thickness and curvature). Then, we opted to split the key phases of the spread in a composite figure (now figure 2). As indicated above in the section « Figures », we took advantage of new functions (including arrows) of the new version of Evolaps. A dated phylogenetic tree has been added to figure 2 (ex figure 3). The dates and their HPDs are given in the text.

The following paragraph has been added to address the issue of the sampling biases (lines 235-253): “ Inference using statistical phylogeographic models, including those that are used in the present study, may be impacted by the way samples were collected (Guindon and Maio, 2021; Kalkauskas et al, 2021). In particular, observations deriving from a single point in time do not convey information about a directionality in the migration patterns, making it difficult, if not impossible, to accurately reconstruct ancestral locations. Biases in sampling locations, whereby these locations follow particular geographic patterns that depart from the distribution of the whole population, may also result in biases in ancestral reconstructions. The latter will reflect sampling locations rather than the spatial distribution of ancient populations. Finally, sampled locations are the product of two processes. The first governs the random fluctuation of lineage locations during the course of evolution. The second corresponds to logistic constraints that prevent specific regions from being included in the sampled areas. Ignoring these constraints leads to overly precise estimates of the spatial diffusion parameters (Kalkauskas et al, 2021). The present study relies however on heterochronous data, which, at least in theory, conveys information about the directionality of the migration patterns. Also, it is likely that most regions of RYMV circulation in East Africa were surveyed and incorporated in our sample. Then, the sampling area should reflect the spatial distribution of the corresponding pathogen’s population. The sampling scheme here is thus clearly different from targeting a small number of specific regions, selected based on logistic considerations only. While sampling biases cannot be fully excluded, their impact on the reconstructions of RYMV spread in Africa should be limited.”

>Kalkauskas, A., Perron, U., Sun, Y., Goldman, N., Baele, G., Guindon, S. and De Maio, N., 2021. Sampling bias and model choice in continuous phylogeography: Getting lost on a random walk. PLOS Computational Biology, 17(1), p.e1008561.

>Guindon, S. and De Maio, N., 2021. Accounting for spatial sampling patterns in Bayesian phylogeography. Proceedings of the National Academy of Sciences, 118(52), p.e2105273118.

*2) The lack of temporal signal in the molecular clock analysis is also perplexing. The root-to-tip regression shows surprisingly little correlation between sample dates and divergence for such a rapidly evolving virus. I'm therefore wondering if other factors may be obscuring the temporal signal here? For example is there unaccounted for recombination? Errors in the sampling dates? Or even possibly variation in the underlying clock rate (beyond what can be accounted for by the relaxed clock model) due some lineages evolving at higher rates? For a seed-borne virus it could be that some viral lineages essentially lie "dormant" while in seed and therefore do not accumulate substitutions at the rate expected for continuously replicating virus. Regardless of what's driving this, it might be worth inspecting the outliers for patterns that could explain this rate heterogeneity.*

A weak to moderate temporal signal in the datasets made of heterochronous sequences of plant viruses is a general feature (Mbewe et al, 2024 [www.sciencedirect.com/science/article/pii/S016817022400090X](http://www.sciencedirect.com/science/article/pii/S016817022400090X) for a recent review), in contrast with the strong temporal structure of several animal or human animal viruses. A strong linear relationship between the sampling date and the distance to the root in a root to tip regression similar to those currently encountered with animal or human animals has not been observed with plant viruses (West Nile Virus: <https://nextstrain.org/WNV/NA?branches=hide&l=clock>;

seasonal flue <https://nextstrain.org/seasonal-flu/h3n2/ha/2y?branches=hide&l=clock>;

enterovirus : https://nextstrain.org/enterovirus/d68/genome?branches=hide&l=clock).

This possibly reflects the lack of a defense system in plants as efficient as the immune system of animal and human viruses. Whatever the causes, this makes the analysis of the temporal signal of plant viruses complex. Both datasets in West and East Africa passed the various temporal tests. So both datasets contain some temporal signal. However, the temporal signal of the West African dataset was stronger than that of the East African dataset. This likely reflects that a regular sampling was conducted over a longer period in West Africa (three decades) than in East Africa (two decades). Interestingly, a recent article reported that substitution rates of plant viruses decreased with the sampling time span (Mbewe et al, 2024). We added a sentence with this reference (lines 187-188) “Interestingly, a recent article reported that substitution rates of the plant viruses decreased with the sampling time span (Mbewe et al, 2024)”. Altogether, although the East African data set does contain some temporal signal, it is too weak to get robust estimates of the substitution rate and node ages including the TMCRA. This is the reason why a prior derived from the West African dataset was introduced.

Among the other factors possibly obscuring the temporal signal, recombination can be excluded as the coat protein gene sequences used were recombinant free. We can also exclude major errors in the sampling dates. None of the sampling dates were extracted from NCBI. All the isolates were collected by the authors of the article, or by scientists which have been directly associated with studies that reported them. The early isolates were regenerated from time to time when the long stability of RYMV isolates in a deep freezer was not fully appreciated. This may have contributed to blurring the temporal signal, especially that of East Africa dataset that contain the oldest isolates. We cannot also fully exclude a variation in the underlying clock rate beyond what can be accounted for by the relaxed clocks due to some lineages evolving at a higher rate. However, inspecting the divergence patterns for outliers in the root-to-tip regressions did not indicate such issues. We thought at the last possibility raised by reviewer 3 «For a seed-borne virus it could be that some viral lineages essentially lie « dormant » while in the seed and therefore do not accumulate substitutions at the rate expected for continuously replicating virus ». This would apply to the situation in East Africa where, as explained by the historical reasons given in the text, the virus survived for long periods associated with the seeds. So the following sentences were introduced under the « Temporal signal » heading in the Result section (lines 188-191): « Viral isolates in contaminated seeds do not accumulate nucleotide substitutions over time. These periods of stasis, both prolonged and frequent during the initial stages of RYMV epidemiology in East Africa (see below), may introduce evolutionary rate variation among viral lineages ».

> Mbewe et al, 2024; doi.org/10.1016/j.virusres.2024.199397

**Part III – Minor Issues: Editorial and Data Presentation Modifications**

*Reviewer #1: Introduction - provides a good general overview of RYMV and its epidemiology. Some of the paragraphs seem to jump around a little, which affects the flow of information. Some slight revision in this section would achieve better flow.*

The following sentences were added to better link the first paragraph of the Introduction to the rest of the text (lines 88-93): “This study aimed at evaluating the relative importance of abiotic and vector-mediated transmission in plant virus species with a dual mode of transmission (i.e. vector and abiotic). We hypothesized that the contribution of abiotic transmission to plant virus spread has been substantially underestimated (Jones 2018; Peters et al, 2024). Rice yellow mottle virus (RYMV), which affects rice crops in Africa, is as an appropriate model to test this hypothesis”.

*Some specific minor points in the Introduction:*

*Line 83: clarify that these genera are in different taxonomic families. The wording now makes it seem like they are genera within a family.*

The families of the genera are now indicated in the revised sentence (lines 82-85) « Owing to their virion stability and high titer, Sobemoviruses (Solemoviridae family), Tobamoviruses (Virgaviridae), Potexviruses (Alphaflexiviridae) and Tombusviruses (Tombusviridae) are listed as candidates for contact and abiotic transmission ».

*Line 87: Again, give the family of the Sobemoviruses.*

Now indicated (see above)

*Line 93: Typo, "reaches"?*
Corrected

*Lines 189-194: Some or all of this seems like it should be in Methods.*
After several attempts to move paragraphs on temporal signal to the Materials and Methods, we opted to leave them at the beginning of the Result section to keep the consistency between the paragraphs and the coherence with other sections of the Results.

*Line 324: the mention of rabies virus seems very out of context. If comparisons are useful to make to this virus that should be done in the Discussion.*

Modified accordingly

*Please use past tense.*
Implemented

*Discussion - It would be really helpful to illustrate the seven periods that you have identified into a figure of sorts, such as a time line or similar.*

Implemented; see above, section « Figure points »

*Reviewer #2: I think the presentation of the paper may be improved, and the suggestions below could be taken into account:*

*1. Check the reference list, as the format is not uniform: Doi is given for some references and page number for other.*

Pages numbers are indicated for articles without doi.

*2. Line 141. Unclear. What means rice cultivation is localized?*

The sentence was modified as follows: « … rice cultivation until the last decades remained confined to lake shores, river banks and wetlands ».

*3. Line 163 and on. Since the Material and Methods comes after the results, it would be good if right at the begging of the results the data sets on which the analyses are based were explained, not just referred as WA335 etc. Also, defined BETS.*

The following sentence has been added to the Materials and Methods section (lines 627-631) to describe BETS. « BETS is a Bayesian evaluation method of the temporal signal which involves comparing the fit to the data of two models: a model in which the data are accompanied by the actual (heterochronous) sampling times, and a model in which the samples are constrained to be contemporaneous (isochronous) (Duchêne et al, 2020) ».

*4. Lines 184-185. I don’t find this information in Table 1.*

Actually, these sentences come from an earlier version of the ms. They should have been removed in the submitted version. We apologize for this mistake. The right paragraph is as follows:

« As the temporal signal from the East African corpus was not sufficient alone to allow a valid reconstruction of the spatio-temporal dispersion of the RYMV in East Africa, priors derived from the WA335 dataset were applied. Accordingly, for the EA335 phylogeographic reconstructions, a prior was specified on the mean rate of the uncorrelated log-normal relaxed molecular clock (the “ucld.mean”parameter), a normal distribution with a mean of 1.24 10^-3^ substitutions per site per year and standard deviation of 1.16 10^-4^. An informative prior was also set on the standard deviation of the uncorrelated log-normal relaxed clock (the “ucld.stdev” parameter), a normal distribution with a mean of 1.09 x 10^-3^ substitution per site per year and standard deviation of 2.87 x 10^-4^ ». We have now also introduced the right version of Table 1.

*5. The Discussion is very long and sometimes repetitive, what diffuses somewhat the message.*

Paragraphs of the Discussion dealing with methodological issues (sample bias, flexibility of the RRW) were moved to the Result or in the Materials and Methods sections. A paragraph on the hypothetical role of positive selection in the differential epidemiological success of the strains, a topic that is outside of the scope of this study, has been omitted in order not to distract from the message. Other paragraphs were shortened.

*6. Also, the wealth of historical data provided contributes to explain, and complements, the hard-core results based on sequence analyses, but still is circumstantial evidence (cannot be otherwise) and should be so presented.*

The following sentence, directly inspired from the comment, has been added at the beginning of the Discussion (lines 390-391) « The historical data provided circumstantial evidence that support, explain and complement the interpretation of the phylogeographic reconstructions ».

*7. Line 358. Eragrostideae (I think this is the correct spelling) is a tribe within the Poaceae not a family. Thus I do not know if the sentence refers to hosts in other tribes of Poaceae or in other families related to Poaceae.*
The sentence has been modified as follows « … resulted in spillovers to cultivated rice, presumably - given RYMV host range - from primary hosts of the Oryzeae tribe of the subfamily Oryzoideae and of the Eragrostideae tribe of the subfamily Chloridoideae within the grass family Poaceae. ».

*8. The Introduction does not indicate that finger millet (whatever it is, non -English-speaking readers would benefit from the botanical name) is a host of RYMV. A reference should be given.*

The sentence has been modified as follows: « Primary hosts also include the finger millet (*Eleusine coracana*) of the subfamily Chloridoideae, a local host of RYMV (Bakker, 1974; Allarangaye et al, 2007), widely cultivated through slash and burn cultivation in these mountains during the 19^th^ century (Paul, 2003). ». The botanical name of teff and the reference to the article indicating that it is a host of RYMV are given in the sentence modified as follows: « The widespread cultivation of teff (Eragrostis tef), a host of RYMV (Bakker, 1974), in the Ethiopian highlands may have facilitated the dissemination ».

*9. Lines 362-363. Change to “ Strains specific to poaceae species….”*

Corrected

*10. Line 381. Dispersal by boat, or rice introduced by boat?*

The sentence has been modified as follows: « … where rice had been introduced at the end of the 19th century along the caravan roads (Koponen, 1988) or by boat (Leclercq, 1913; Tosh, 1970).”

*11. Genomic regions used in analyses are sometimes identified by their product (e.g., coat protein sequences) and sometimes by the ORF (e.g., ORF4). As not everybody is familiar with the genome organization of this virus, it would help if some explanation was given on the non-CP regions analysed.*

The following paragraph has been added (lines 571-577) « The genome of RYMV is organized into five overlapping open reading frames. ORF1, located at the 5' end, encodes a protein involved in virus movement and gene silencing suppression. ORFx overlaps with the 5' end of ORF2a and is translated through a leaky scanning mechanism. ORF2a and ORF2b are overlapping ORFs that encode the central polyprotein. ORF2a encodes a serine protease and the Vpg. ORF2b translated through a -1 ribosomal frameshift encodes the RNA-dependent RNA polymerase. ORF4 located at the 3' end encodes the coat protein. This ORF is expressed via a subgenomic RNA (Somera et al, 2021). »

*Reviewer #3: Author summary: "It also highlights the role of human transmission of pathogens, even vector-borne, and sheds light on the risk of transmission of RYMV and of other plant viruses from Africa to other continents." -- I wonder why the authors qualified this statement with "even vector-borne"? To me it seems like humans have played a massive role in the spread of many vector-borne viruses.*
The sentence has been removed.

*Introduction: The first paragraph seems a bit out of place. I was left wondering why I was being provided with all this information about transmission through direct contact when RYMV is (mostly?) vector-borne.*
The sentence « The sobemovirus species differ... by the mode of transmission, most often by beetles » was misleading. It does not mean that sobemoviruses are mostly transmitted by beetles but that beetles are the vectors most frequently listed in sobemovirus transmission (compared to aphids for instance). The sentence was modified as follows: “The sobemovirus species differ from the three other genera by having a narrow host range and by being transmitted by insect vectors. Beetles are the vectors most frequently listed in sobemovirus transmission”.

*Lines 105-106: "Altogether, contact and soil tranmission accounts for on-site survival of the virus and its local spread." -- Could you please clarify how this relates to vector-borne transmission? Can the virus persist without vector transmission?*

As tentatively explained in this paragraph, observations and experiments gradually show the importance of contact transmission in RYMV epidemiology. Transmission by beetles is an alternative mode of dispersal of the virus, possibly a minor one, as suggested by the situation in East Africa explored in this study.

*Lines 130-131: "the phylogeographic reconstructions are not affected by the time dependent rate phenomenon" -- This does not seem like a defensible statement. Surely movement rates between different locations must to some extent fluctuate through time.*

The sentence was misleading. It originated from a paragraph of reference 24 (Ghafari et al, 2024; reference listed) « Over shorter timescales, we found that the TMRCA of the two most divergent RYMV isolates (RYMV-1 and -2 in Fig 3A) was 156 (95% HPD: 80–288) years BP, in agreement with previous estimates obtained from standard Bayesian molecular clock analysis which do not correct for Time Dependent Rater Phenomenon TDRP. This finding further suggests that saturation over such short timescales is unlikely to have significant impact on TMRCA estimates of sobemoviruses ». It indicates that the TMRCA of the phylogenetic reconstructions of RYMV are not impaired by the TDRP. As far as we know, this has never been checked in the phylogeographic reconstructions of any plant viruses. This is the reason why we pointed it out. We modified the sentence as follows: « the timescale of the phylogeographic reconstructions is not affected by the time dependent rate phenomenon ».

*Line 163: "BETS" - please define BETS. Also in the same line its not clear what "increase in sample size" the authors are referring to.*

This point was also raised by Reviewer #2 (see above).

The sentence was modified as follows: « BETS analyses indicate that the increase in the sample size (from 261 to 335 sequences for the West African dataset, and from 240 to 335 sequences for the East African sataset) lead to a stronger temporal signal in each dataset ».

*Finally, for the comparative dispersal analysis, Trovao et al. (2015) previously performed an extremely elegant analysis examining why RYMV's diffusion rates vary through continental Africa and linked it to the intensity of rice production. I find it somewhat odd that these results where not discussed here. Disclosure: I have no affiliation with the Trovao et al. paper. I just think it deserves revisiting here.*

The following paragraph has been added in the Discussion (lines 514-526): « Given a host range that is restricted to rice and closely related poaceae species, a direct connection between RYMV epidemiology and rice ecology was anticipated (Traoré et al, 2009). However, elucidating the precise nature of this relationship has proven challenging. Linear relationships between the intensity of rice cultivation and the transition frequency of RYMV lineages in West-Africa have been proposed (Trovão et al, 2015), as well as between rice cultivation intensity and virus population size in the irrigated valley of Niger (Issaka et al, 2021). Nevertheless, further statistical analyses failed to provide supports for such associations between the dispersal dynamic of viral lineages or virus population size and rice-cultivated areas (Dellicour et al, 2018; Issaka et al, 2021). Factors that make the relationship between RYMV epidemiology and its hosts more complex than initially proposed in early modelling attempts have been explored (Rakotomala et al, 2019 ; Issaka et al, 2021). Interestingly, in East Africa, the transfer of rice seeds, rather than rice cultivation was critical in RYMV dispersal. Virus spread in East Africa was more closely associated with human-mediated long-distance transport of rice seeds than with the short-range vector-borne movements typically linked to rice cultivation ».
