# Supplementary material for: Grains, trade and war in the multimodal transmission of Rice yellow mottle virus: An historical and phylogeographical retrospective
Source: PLoS Pathog. 2025 Jun 17;21(6):e1013168. doi: 10.1371/journal.ppat.1013168 (PMC12173394; doi:10.1371/journal.ppat.1013168)
Supplement: Attachment — Submitted filename: Rebuttal letter_250422.docx [file ppat.1013168.s008.docx]

Dear Pr Malmstrom, dear Carolyn,

Thank you for editing the revised version of our ms and for your encouragements. Accordingly, the ms was modified as followed:

*--"BETS analysis" still appears in the Results before it is defined/introduced.*

This is the consequence of the position of the “Materials and Method” section after the Discussion (and subsequently after the Result section). So we moved the following sentences from the “Materials and Methods” section to the “Result” section:

“BETS is a Bayesian evaluation method of the temporal signal which involves comparing the fit to the data of two models: a model in which the data are accompanied by the actual (heterochronous) sampling times, and a model in which the samples are constrained to be contemporaneous (isochronous).BETS analyses indicate that the increase in sample size of heterochronous sequences leads to a stronger temporal signal in West Africa (from 261 to 335 sequences) and in East Africa (from 240 to 335 sequences) (Table 1)”.

*--Lines 240-241: It might be helpful to note that the historical scenario described below is the most probable given the phylogeographic reconstruction - which would still acknowledge that there is some uncertainty in this reconstruction.*

To accommodate that comment from Reviewer 3, we changed the sentences:

“The spatio-temporal dispersal of RYMV lineages in East Africa was reconstructed from the capsid protein gene sequences of 335 isolates (Fig. 2). The following periods are distinguished.”

into this single sentence:

“The spatio-temporal dispersal of RYMV lineages in East Africa was reconstructed from the capsid protein gene sequences of 335 isolates (Fig. 2), giving rise to the following most probable historical scenario.”

In our opinion, there is no need to add anything else as this implicitly acknowledges the uncertainty as the Reviewer 3 points out.

*--The two additional points made by reviewer 3 about whether material should be in Discussion or elsewhere are helpful points but I think the ms. can stand as is on those, so you need not respond to these, although it is fine if you do so.*

As indicated in the Rebuttal letter of the revised version, we have made various attempts to address these points. We prefer to keep them as they stand as you leave us the possibility.

--In the Acknowledgement section, we added the following sentence: “We would like to express our sincere gratitude to the guest editor and to the three reviewers for their time and contribution to the article”.

--The following reference was added:

Bovill EW. 1917. Notes from East Africa: The Uluguru Mountains and the Rufiji Plain

The Geographical Journal, Vol. 50, 4, 277-283.

--A few typo were corrected and a few words were edited.

Best Regards
